# Analysis of the Protective Potential of the Amniotic Membrane in an *In Vitro* Experimental Model of Demyelination in Mouse Brain Organotypic Slices

**DOI:** 10.1021/acsomega.5c02999

**Published:** 2025-07-23

**Authors:** Melissa Guimarães, Gabriela A. T. Calheiro, Luciana B. Sant’Anna

**Affiliations:** Laboratory of Histology and Regenerative Therapy, Research and Development Institute, 67655 University of Vale of Paraíba, São José dos Campos 12244-390, Brazil

## Abstract

Amniotic membrane (AM) is a biological material recognized for its regenerative, anti-inflammatory, and immunomodulatory properties, constituting a promising approach for the treatment of neurodegenerative diseases, such as demyelinating diseases. Some neurodegenerative diseases, such as Multiple Sclerosis (MS), occur with demyelination, which is a process characterized by the loss of myelin, a structure responsible for the adequate conduction of nerve impulses, compromising neuronal functionality. In this context, this study aimed to investigate the efficacy of AM in protecting nervous tissue against the demyelinating effects of lysophosphatidylcholine (LPC, lysolecithin), using organotypic brain slices from C57BL/6 mice as an *in vitro* experimental model. Four experimental groups were established: C–H (healthy slices), C-DEM (slices demyelinated with LPC), C-AM (healthy slices with AM), and AM-LPC (slices protected by AM before LPC). The analyses included histological staining (Hematoxylin and Eosin, Luxol Fast Blue), metabolic test with 2,3,5-triphenyltetrazolium chloride (TTC), and Scanning Electron Microscopy (SEM). Results showed that AM preserved myelin and tissue architecture in the challenged slices, while the demyelination group presented microcavitations, structural disorganization, and loss of distinction between white and gray matter. The TTC assay revealed high metabolic activity in the slices protected by AM, in contrast with the low activity in the demyelinated group. SEM analysis reinforced the efficacy of AM, evidencing a preserved organization of the brain parenchyma in slices protected by AM. Thus, the results demonstrate that AM was effective in protecting nervous tissue against the demyelinating effects of lysophosphatidylcholine, preserving myelin, structural organization, and metabolic activity of brain slices, as evidenced by histological, metabolic, and ultrastructural analyses with SEM.

## Graphical abstract

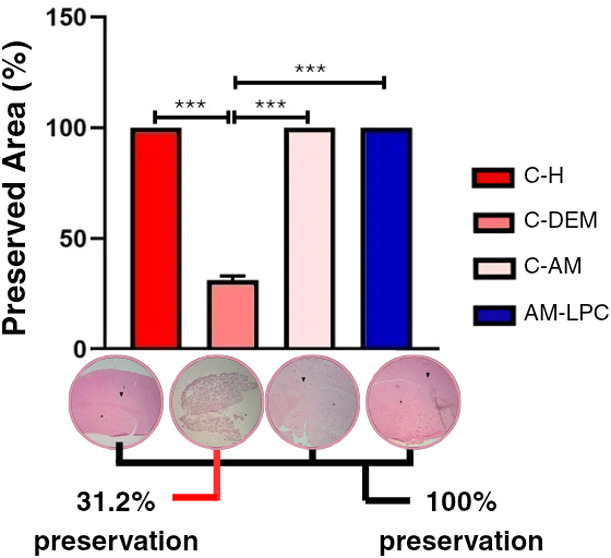

## Introduction

The Central Nervous System (CNS) regulates motor, sensory, and cognitive functions, ensuring the interaction of organisms with the environment. Composed of the brain, spinal cord, and optic nerve, it processes information and coordinates vital functions. The brain and spinal cord are composed of white and gray matter. Gray matter is composed primarily of neuronal cell bodies, and white matter is characterized by a high concentration of myelinated axons, the integrity of which is essential for the conduction of nerve impulses. Changes in myelin can compromise this functionality and lead to significant neurological deficits.^1–3^

Demyelination is characterized by the loss of myelin, impacting neural transmission and resulting in functional deficits.^4,5^ MS is one of the main demyelinating disorders, leading to fatigue, muscle weakness, vision problems, and cognitive deficits.^6,7^ With population aging, neurodegenerative diseases become more prevalent, requiring effective therapeutic strategies to minimize their social and economic impacts.^8,9^

Experimental models, such as organotypic brain slices, are widely used to study demyelination and to test regenerative therapies. This model maintains the three-dimensional organization of the tissue and allows the study of pathophysiological processes.^10^ Brain slices are cut between 200 and 500 μm and cultured on porous polytetrafluoroethylene (PTFE) membranes, which allow for the air–liquid interface and the penetration of therapeutic agents.^11^ Lysolecithin is a demyelinating agent widely used in these studies to induce controlled demyelination.^12,13^

Amniotic membrane (AM) has emerged as a promising approach in regenerative medicine. It is the innermost layer of human fetal membranes, rich in mesenchymal stem cells and amniotic fluid, which have therapeutic potential.^14–16^ Its extracellular matrix (ECM) contains collagen, laminin, proteoglycans, glycoproteins, elastin, and fibronectin, providing mechanical strength and flexibility.^17^ In addition, AM releases bioactive factors that promote regeneration, have anti-inflammatory and immunomodulatory properties, favoring tissue integrity.^18–21^

Studies demonstrate that AM reduces hepatic fibrosis in experimental models,^22^ accelerates the healing of skin wounds,^23^ and promotes the regeneration of peripheral nerves^24,25^ and the spinal cord.^26^ Its immunomodulatory properties are attributed to the expression of proteins such as HLA-G and TGF-β, which suppress immune responses and favor immunological tolerance.^27^ Soluble factors released by AM inhibit the proliferation of T cells and promote the differentiation of regulatory T cells, inhibiting Th1 and Th17, related to MS progression.^28,29^

Given its therapeutic potential, this study proposes the use of AM as an approach to protecting organotypic brain slices from lysolecithin-induced demyelination. It is expected that its ECM, rich in collagen and elastin, can confer mechanical resistance capable of acting as a barrier against the penetration of exogenous agents, such as lysolecithin. In addition, its bioactive properties are considered to modulate inflammatory responses and provide growth factors that preserve myelin and stimulate cell regeneration.^17^

The application of AM in demyelination models represents an innovative strategy to investigate its ability to preserve myelin. There are no previous studies applying this membrane in this experimental model to evaluate its protective potential. Thus, this study aims to explore AM as a therapeutic tool in the context of lysolecithin-induced demyelination in organotypic brain slices. This approach may open new perspectives for the treatment of demyelinating diseases, contributing to improving the quality of life of patients affected by these conditions.

## Materials and Methods

### Obtaining the Placenta and Preparing the Amniotic Membrane

This work was approved by the Research Ethics Committee (CEP), under protocol number 6.389.780. After approval by the CEP, 10 full-term human placentas, with a gestational age equal to or greater than 39 weeks, were obtained from elective cesarean sections of women with normal pregnancies, at the maternity ward of the Hospital Santa Casa de São José dos Campos, after maternal consent by signing a Free and Informed Consent Form, and after certification of negative results for Hepatitis B and C, syphilis, and HIV-1 and 2.

The AM was transported to the laboratory at the Research and Development Institute under refrigeration at around 10 to 15 °C, where, under sterile conditions, it was manually separated from the chorionic membrane and washed extensively in physiological saline solution containing 100 U/mL of penicillin, 100 μg/mL of streptomycin, and amphotericin B. The AM was cut into fragments of adequate size (2 × 2 cm), and thickness between 0.02 and 0.1 mm, and marked to allow identification of the mesenchymal face that will be placed in the wells of the plate of 6 wells to cover the brain slices that were cultured in the wells. The pieces were stored separately at room temperature in 50 mL vials containing DMEM culture medium without serum and without phenol red under sterile conditions, until application.^22,30^

### Animals and Experimental Groups

Eight C57BL/6 mice were used to conduct the experiments, with prior approval from the Animal Use Ethics Committee (CEUA), under protocol number A3CEUA/2023.

The mice were purchased from CEMIB (Multidisciplinary Center for Biological Research in the Area of Science in Laboratory Animals) of the State University of Campinas (UNICAMP). The animals were transported to São José dos Campos in appropriate boxes, equipped with apples to prevent dehydration during the journey. Upon arrival in São José dos Campos, the mice were housed in the Bioterium of the Research and Development Institute (IP&D) of the University of Vale do Paraíba (UNIVAP) in microisolators placed in ventilated racks, with three animals per unit. During the housing period, they received water and food *ad libitum*, in addition to rolls of toilet paper, as environmental enrichment. The room temperature was maintained at approximately 22 °C, with a 12-h light/dark cycle.

The animals were used to obtain brain slices, which were divided into different experimental groups: Healthy Control Group (C–H); Demyelination Control Group (C-DEM); Membrane Control Group (C-AM); and Membrane LPC Group (AM-LPC). The C–H referred to the group whose organotypic slices did not undergo the demyelination process and did not receive AM application on the slices. In the C-DEM Group, the organotypic slices were demyelinated with LPC, but they did not receive AM application. In the C-AM Group, the organotypic slices were not demyelinated but received AM, which was positioned on top of the slices. Finally, in the AM-LPC Group, three AM fragments were positioned over the brain slice, followed by the application of LPC directly onto the slice protected by AM. The objective was to evaluate the efficacy of AM in protecting the tissue against demyelination induced by the demyelinating agent. Thus, AM was challenged with LPC to determine its ability to act as a protective barrier, preserving the integrity of the myelin sheath under the proposed experimental conditions. Each of the 3 analyses was performed in triplicate.

## Experimental Section

### Procedures for Obtaining and Cultivating Organotypic Brain Slices

In the following sections that describe the steps for obtaining and cultivating organotypic brain slices, the methodology used for the culture of organotypic brain slices will be presented. The methodology was based on protocols previously established in the literature,^31,32^ with some adaptations to adjust to the objectives and experimental conditions of the study.

### Preparation of Culture Media and Supplies for Brain Dissection

Before the mouse brain slices were obtained, solutions for obtaining and culturing the slices were prepared. Initially, the dissection medium was prepared, consisting of MEM (Minimum Essential Medium, NovaBio, Brazil) culture medium and 1% antibiotic (penicillin and streptomycin, Sigma, St. Louis, MO). This medium, also called dissection buffer, was used to prepare and manipulate the tissue slices before they were placed on a culture plate. Next, the MEM culture medium was prepared supplemented with 25% fetal bovine serum (Sigma, St. Louis, MO), 25% Earle’s salts (NovaBio, Brazil), 2.6 mg/mL of 45% glucose (Exodo Cientifica, Brazil), 1% glutamine (Exodo Cientifica, Brazil), and 1% antibiotic (penicillin and streptomycin). Using sterile forceps, an organotypic insert were placed in each well of a 6-well plate, ensuring no bubbles are trapped under the membrane. One mL of supplemented MEM was pipetted into each well of the plate. Afterward, the plate was placed in an incubator at 37 °C and 7.5% CO_2_ to be heated for at least 2 h before dissection.

### Procedures for Obtaining Brain Slices

The procedures for obtaining and dissecting the slices were performed in a sterile laminar flow hood. The tissue chopper was prepared by placing a new blade and then pipetting 300 μL of 70% ethanol under the cutting stage to ensure that the sample remained in place. The tissue chopper blade was gently cleaned with a cotton swab and subsequently dried before being used. All dissection tools were sprayed with 70% ethanol and dried before dissection. Next, two 10 cm Petri dishes were prepared: one with 15 mL of supplemented MEM and the other with 10 mL of dissection medium. This medium was oxygenated with 95% oxygen and stored on ice for 15 min before the dissection began.

To obtain the brain slices, the mice were sedated with a combination of xylazine, ketamine, and fentanyl, followed by general anesthesia with thiopental. After confirmation that the animals had achieved an adequate plane of anesthesia, a booster dose of thiopental was administered to ensure a deep plane of anesthesia and the onset of significant cardiorespiratory depression. After ensuring that the animals were deeply anesthetized and ensuring that they were in a state of complete hypnosis and unconsciousness, intracardiac perfusion with potassium chloride was performed to induce cardiac arrest.

After euthanasia, the animals were decapitated and the skull over the forebrain and cerebellum was rapidly removed with a sagittal cut followed by a lateral incision. The cranial nerves were cut from the ventral surface of the cerebrum and cerebellum, carefully displacing them to the side. The cerebrum and cerebellum were placed in a sterile 35 mm dish containing dissection medium. Using a scalpel blade, the cerebellum was separated from the forebrain. The hemispheres were then separated by a midsagittal cut in the forebrain and cerebellum. To allow better visualization of the white matter for histological analysis, the hippocampus was removed, leaving only the cerebral cortex with the white matter more internally.

Using a spatula, the brain was transferred to the cutting stage of the tissue chopper with the tissue resting on the rostral aspect of the hindbrain and the caudal aspect of the hindbrain facing the researcher. Using a P200 pipet, excess medium around the tissue was removed, ensuring that the tissue remained moist but not floating in liquid. The tissue was then cut into slices with a thickness of 400 μM on a tissue chopper. After cutting, 100 μL of supplemented MEM medium was pipetted under the tissue so that the slices would float under the cutting table and then be transferred to the previously separated Petri dish containing supplemented MEM medium. Using a spatula and a fine brush, the organotypic slices were carefully separated and subsequently transferred to the organotypic insert positioned in the wells of the plate, containing supplemented MEM, whose plate had been previously incubated in the incubator to be heated. A brain slice was placed in each insert present in the well of the plate.

### LPC-Induced Demyelination and Amniotic Membrane Application

The demyelinating agent used was L-α-Lysophosphatidylcholine from egg yolk (Sigma, St. Louis, MO). Initially, LPC was diluted to a concentration of 100 mg in 0.8 mL of sterile phosphate-buffered saline (PBS, Sigma, St. Louis, MO). The solution was aliquoted into Eppendorfs tubes containing 80 μL each and stored at −20 °C. At the time of the experiment, the stored LPC was diluted in supplemented MEM until reaching a final concentration of 5 mg/mL. In the C-DEM group, the demyelination process was performed using two strategies: (1) direct application of 100 μL of LPC on the organotypic slices; and (2) positioning a small piece of high-absorption paper (1 × 1 cm), soaked in LPC, on the slices. After application of LPC and placement of the soaked paper, the slices were incubated in MEM supplemented at 37 °C with 7.5% CO_2_ for 30 min to ensure sufficient time for LPC to promote demyelination.

In the AM-LPC, before application of LPC, three AM patches were positioned on the organotypic slices, each cut to approximately 1 × 1 cm. Then, the same demyelination strategies employed in the C-DEM Group were repeated, including the application of high-absorption paper soaked with LPC over the AM patches covering the organotypic slices. The slices were then incubated in MEM supplemented at 37 °C with 7.5% CO_2_ for 30 min to ensure uniformity in the experimental procedure.

### Maintenance of Control Groups without Demyelination

In the C–H group, the slices were cut in the previous step and directly positioned on the organotypic inserts placed in the wells of the plate, being incubated with supplemented MEM at 37 °C in 7.5% CO_2_ ^31,32^ for 30 min, to follow the experimental procedure performed with the other groups.

In the C-AM group, the slices were also cut in the previous step, positioned on the organotypic inserts, and 3 AM patches (1 cm × 1 cm) were positioned on top of the slices. However, they were not challenged with LPC to promote demyelination since the objective of this experimental group was to verify whether AM alone promoted any harmful or beneficial action for the organotypic slices. In the same way as performed with the other experimental groups, the slices were also incubated with supplemented MEM at 37 °C in 7.5% CO_2_, for 30 min.

After 30 min of incubation, the slices from all experimental groups were carefully removed from the inserts with the aid of a fine brush and a spatula and were intended for the analyses proposed in this study, with 3 slices for each analysis in each of the 4 groups: histological analysis, SEM, and evaluation of metabolic activity using 2,3,5-triphenyltetrazolium chloride (TTC).

### Histological and Quantitative Image Analysis

After the incubation period, the brain slices were fixed in 10% buffered formalin (Synth, São Paulo, Brazil) at 4 °C for 24 h, subjected to routine histological processing, and embedded in paraffin (Paraplast, Oxford, St. Louis, MO, USA). Semiserial, 5-μm-thick histological sections were obtained using a semiautomatic microtome (Leica RM2245) and stained using two staining techniques: hematoxylin and eosin (HE) and Luxol Fast Blue (LFB) (Sigma, St. Louis, MO), a special stain that stains myelin sheaths blue. The stains were quantitatively evaluated by histomorphometric image analysis to obtain the relationship between the demyelinated areas and the preserved areas of the nervous tissue of the brain slices (occupied by myelin) using ImageJ (National Institutes of Health, Bethesda, MD, USA), an open-source image analysis software. Microscopic images of the stainings were captured with a digital video camera at 6120 × 8160 pixels, 8 bits, coupled to an Olympus binocular optical microscope. All images were cropped to focus on the region of interest, standardized to a size of 4841 × 3095 pixels, and then evaluated in ImageJ to quantify the areas with microcavitations formation in relation to the total image area.

Before the quantification of the demyelinated areas in the ImageJ software was started, the program was calibrated based on a known reference measurement, allowing the precise conversion of the image pixels into real units of measurement. For this calibration, a calibration slide with regular intervals was used in which each division corresponded to 1 μm. After calibration, the demyelinated areas in the images were quantified in pixels and subsequently converted to metric units, ensuring the accuracy of the analyzed data and minimizing possible variations resulting from differences in the scales of the acquired images.

Because some samples did not fit into a single image, even using the smallest available objective (4x), it was necessary to superimpose two photographs to compose a complete image of the sample. This unified image allowed for the calculation of the total area of the slice and the demyelinated areas. Subsequently, the image of the complete slice was loaded into the ImageJ software, where the “Freehand Selections” tool was used to make manual selections of the areas of interest to be quantified.

### Scanning Electron Microscopy (SEM)

After the brain slices were cultured on a plate, they were immediately sent for SEM analysis. For this purpose, each slice intended for SEM was removed from the organotypic inserts and directly positioned in a 24-well plate, where they were immersed in different solutions present in each well of the plate, which comprised the protocol for SEM analysis. The slices remained immersed in each solution for 10 min. Initially, they were immersed in fixative (2.5% glutaraldehyde +2.5% paraformaldehyde +0.05 mol cacodylate buffer), followed by 50% alcohol, 75% alcohol, 100% alcohol, and, finally, in a mixture of hexamethyldisiloxane (HDMS) (Sigma, St. Louis, MO) with 100% alcohol (1:1). After drying at room temperature for 12 h, the samples were coated with gold (10 nm) by Emitech k550x metallizer and mounted on a stub with help of carbon tape. Finally, the samples were sputter coated with gold and analyzed using ZEISS EVO MA 10 scanning electron microscope.^32,33^

The images obtained by SEM were analyzed in ImageJ software to measure surface roughness and fissures in the parenchyma. To evaluate surface roughness, the variation of intensity in the gray scale was used as a parameter. Initially, the images were opened in the program and converted to an 8-bit format (Image > Type > 8-bit), transforming them into a gray scale, where each pixel presents an intensity value between 0 (black) and 255 (white). For the analysis, a region of interest (ROI) representative of the surface was selected using the selection tool, and the measurement parameters were adjusted to include mean, standard deviation, and area (Analyze > Set Measurements), with data collection performed in Analyze > Measure. The standard deviation value of the pixel intensity represents the variation in the intensity of gray tones, serving as an indirect estimate of the surface roughness of the sample. The greater the dispersion of these values, the greater the topographic variation present in the analyzed region.

To evaluate the fissures, the variation in pixel intensity in the grayscale image was used as a parameter, using the thresholding tool (Threshold) in ImageJ. The images obtained by SEM were converted to 8-bit format (Image > Type > 8-bit), allowing manipulation in grayscale. Next, the manual threshold function was applied (Image > Adjust > Threshold), defining the lower and upper intensity limits in order to specifically highlight the darker regions of the image, compatible with fissures areas. The minimum threshold value was empirically adjusted to 70 (with an upper limit of 255), which was the range that best highlighted the fissures, as observed visually. After thresholding, binarized images with the highlighted fissures were generated and saved for later quantification. Additionally, the topographic visualization tool “Interactive 3D Surface Plot” (Analyze > 3D Surface Plot) was used, which generates a three-dimensional graph based on the intensity of the pixels in the original image. Variation in the distribution and height of the peaks was observed, the regularity of which reflects the degree of uniformity of the surface, in addition to the presence of dark and deep regions, interpreted as areas of depression in the sample topography.

### Evaluation of Metabolic Activity of Slices

To assess the metabolic activity of the slices, a metabolic dye called 2,3,5-triphenyltetrazolium chloride (TTC, Neon Analytical Reagents, Suzano, SP) was used, which has the function of indicating cell viability. The principle of this analysis is the reaction of TTC with active enzymes in the mitochondria, intensely staining the areas of metabolically active (viable) tissues with red and staining the areas of nonviable tissues with weaker or absent staining, where cell metabolism is compromised. Initially, the TTC powder was diluted in PBS until a concentration of 2% was reached and was used immediately after preparation. The brain slices were submerged in the TTC solution for 10 min at 37 °C and kept protected from light to prevent degradation of the dye. The slices were then fixed in 4% paraformaldehyde (PFA, Sigma, St. Louis, MO) for a minimum period of 24 h.^34^ After the TTC staining and the fixation period with PFA, the stained slices were placed on a white background, and the images were captured by using a digital camera.^35^

The staining intensity of the TTC-stained samples was analyzed by using ImageJ software. The images were initially opened in the program, and then, the ROI was manually selected, covering exclusively the region of the brain tissue analyzed. Then, the Analyze > Histogram function was used, and the Mode parameter of the red channel was recorded as a representative measure of the predominant shade of red staining. The values of the red channel range from 0 to 255, with lower values indicating more intense red staining (greater tissue viability), while higher values correspond to less red shades (lower viability). Thus, the intensity of the red staining was quantified objectively, allowing a comparison between the different experimental groups.

### Statistical Analysis

The data from the preservation areas obtained by quantitative image analysis are reported as mean ± standard deviation (SD). First, the data were submitted to the Shapiro-Wilk normality test and then, to the parametric test ANOVA followed by the Tukey’s multiple comparisons test. The value of *p* < 0.001 was considered statistically significant (***). Data analysis and graphing were performed using GraphPad Prism software, version 5.00 (GraphPad Software, CA, USA).

## Results

### Histological Analysis

The histological analysis of the organotypic slices of the C–H group (Figures 1A and 2A) stained with HE and LFB showed an organized brain parenchyma, with preservation of the cortical layers and a clear distinction between gray matter and white matter, indicating the structural and functional integrity of the slices, in addition to ensuring their viability for the study. In the region corresponding to white matter, axons were observed in longitudinal and transversal sections (Figures 3, 4), confirming the identification of this area as white matter.

**Figure 1:**
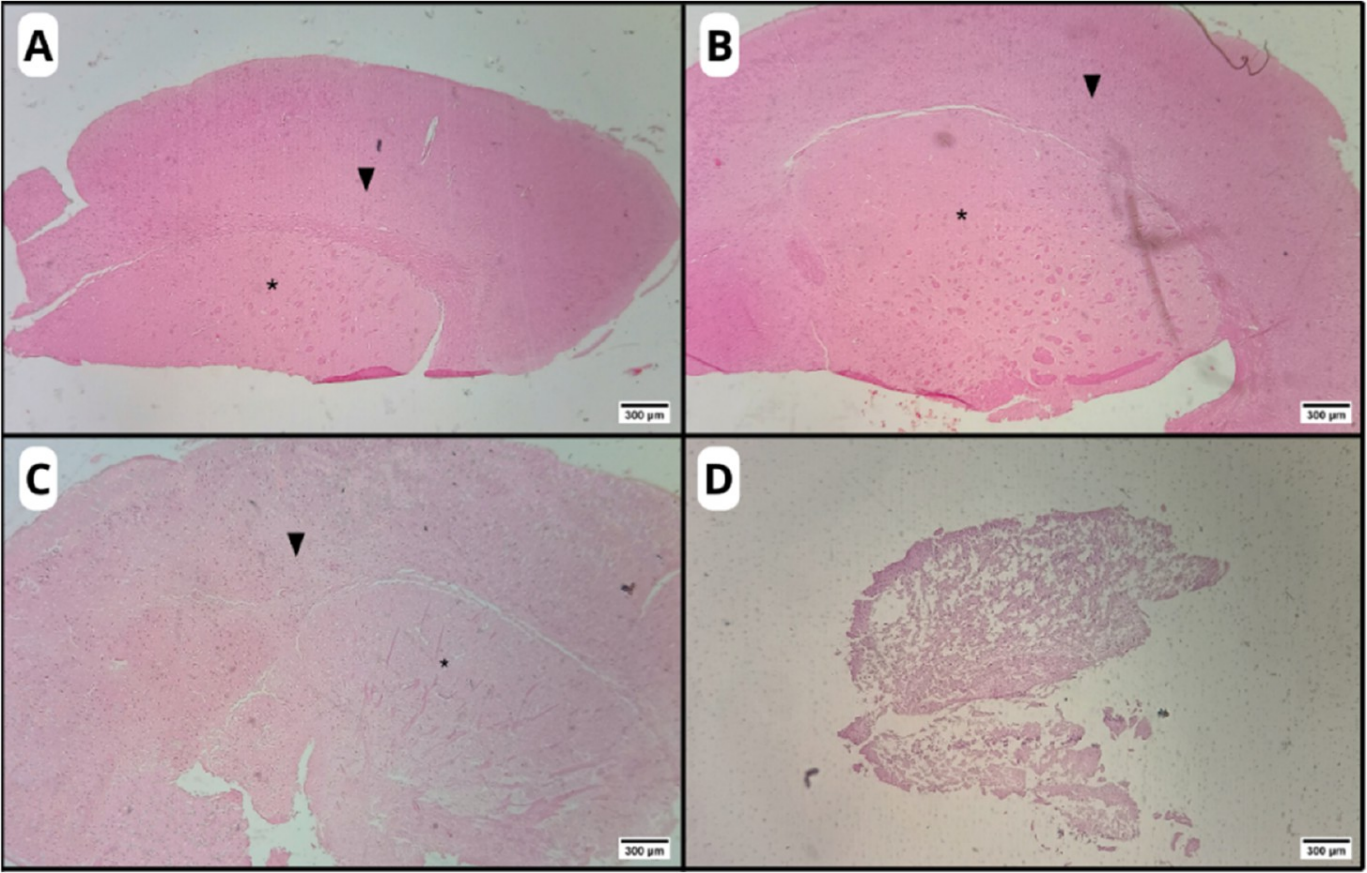
Photomicrographs of the organotypic brain slice in the experimental groups: C–H (A), C-AM (B), AM-LPC (C), and C-DEM (D). Preserved distinction between gray matter (arrowhead) and a white matter-rich region of the striatum (asterisk), observed in all groups, except for the C-DEM group (D), where this differentiation is compromised. The corpus callosum, a classical white matter structure, is also visible. HE staining. Original magnification: 4×.

**Figure 2:**
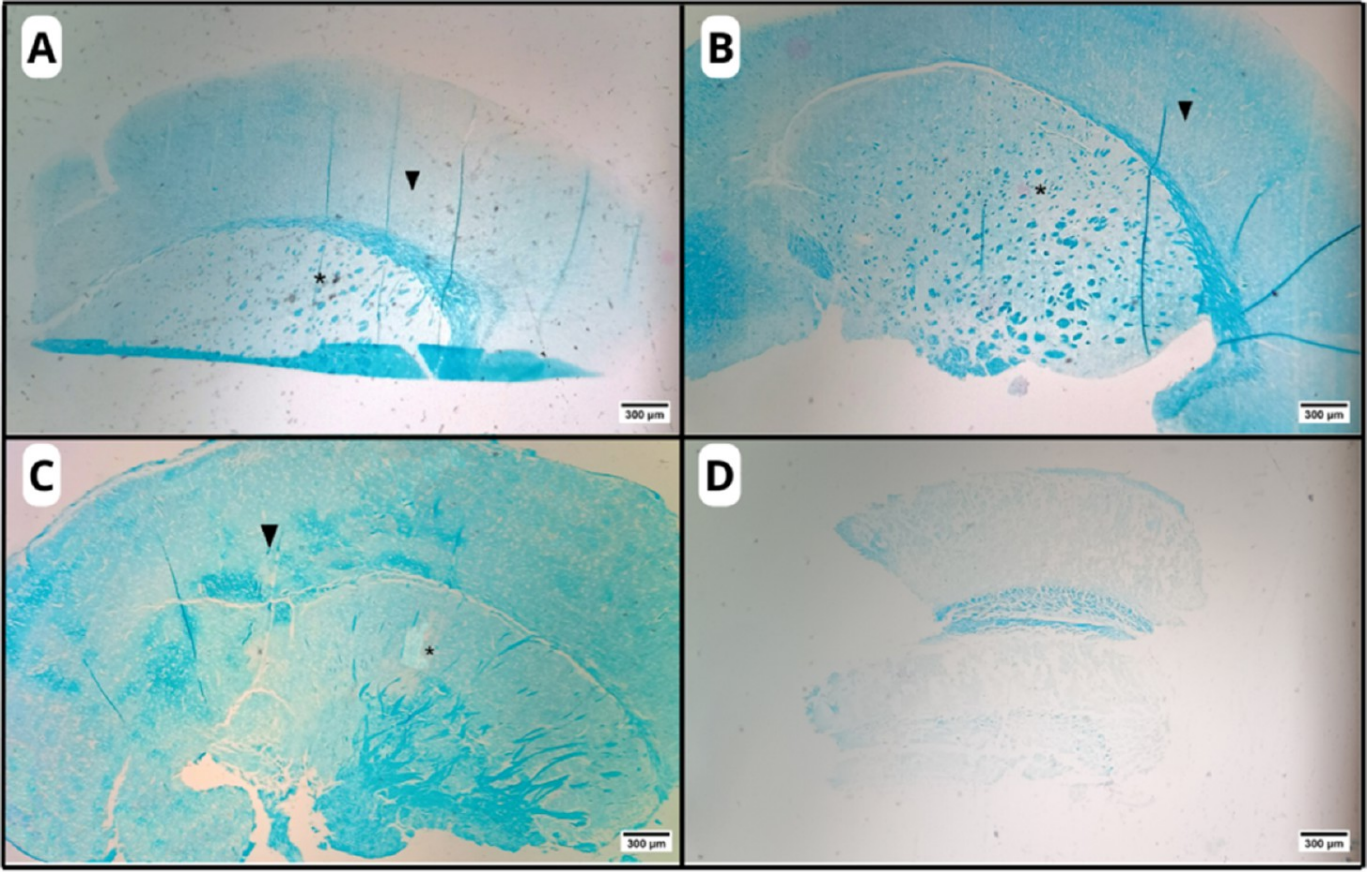
Photomicrographs of organotypic brain slices in the experimental groups: C–H (A), C-AM (B), AM-LPC (C), and C-DEM (D). Preserved distinction between gray matter (arrowhead) and a white matter-rich region of the striatum (asterisk), observed in all groups, except for the C-DEM group (D), where this differentiation is compromised. The corpus callosum, a classical white matter structure, is also visible. LFB staining. Original magnification: 4×.

**Figure 3:**
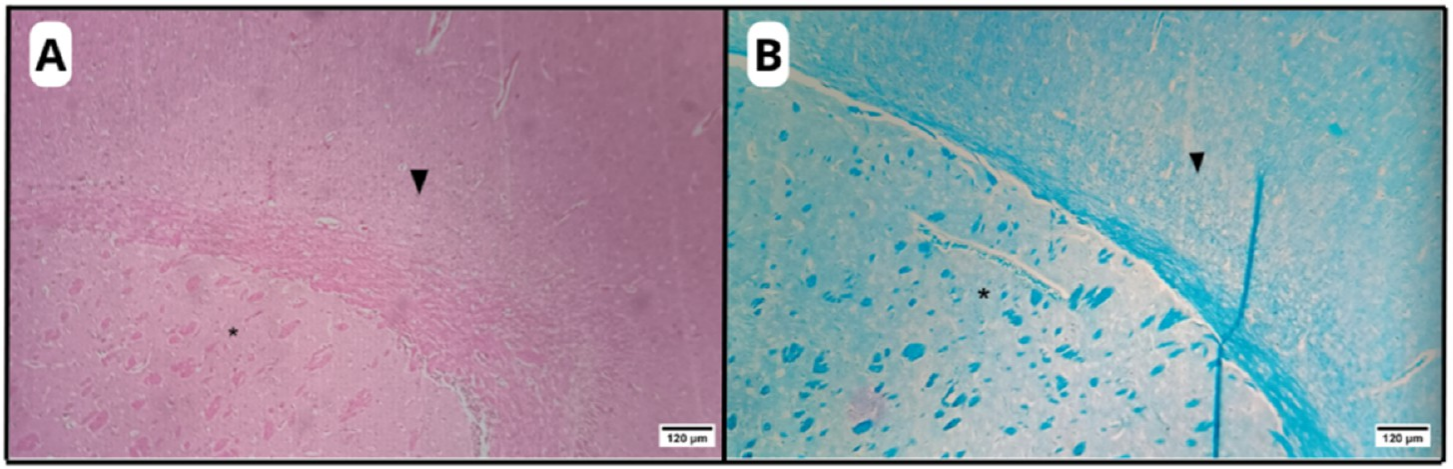
Photomicrographs of organotypic brain slices highlighting the preserved distinction between gray matter (arrowhead) and a white matter-rich region of the striatum (asterisk) observed in the C–H, C-AM, and AM-LPC groups. The corpus callosum, a classical white matter structure, is also visible. HE (A) and LFB (B) stainings. Original magnification: 10×.

**Figure 4:**
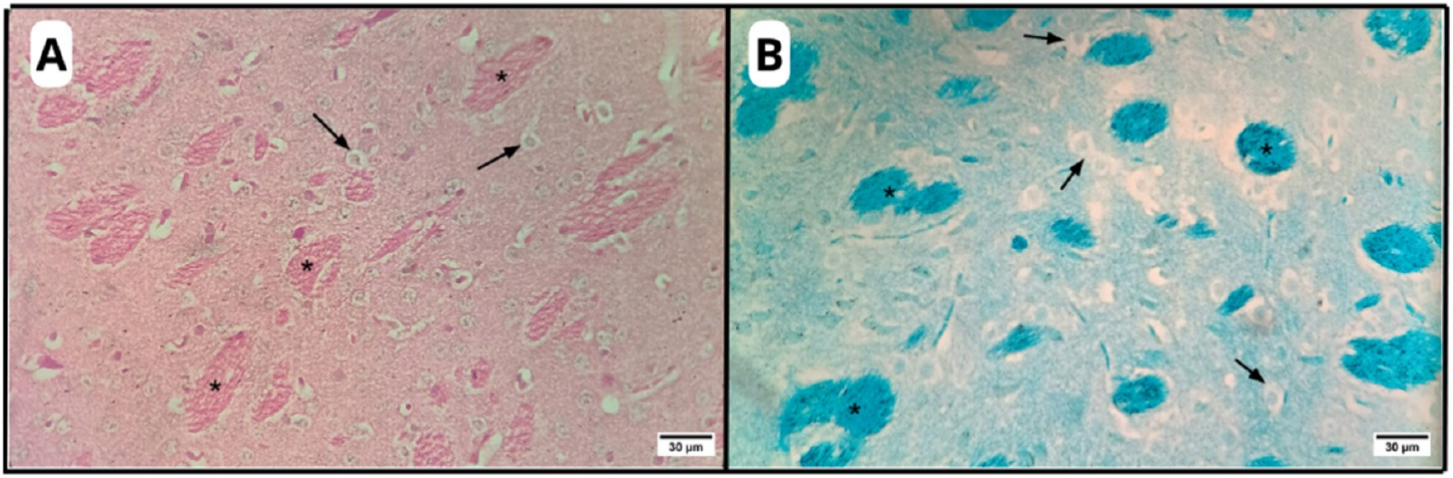
Photomicrographs of organotypic brain slice in the C–H, C-AM and AM-LPC groups, highlighting the white matter-rich region of the striatum: longitudinal (asterisk) and transverse (arrows) axons. HE (A) and LFB (B) stainings. Original magnification: 40×.

Similarly to C–H, the C-AM group (Figures 1B and 2B) also showed preservation of the structural organization of the brain parenchyma, with well-defined cortical layers and a clear distinction between gray matter and white matter, with no evidence of microcavitations or damage to the integrity of the slices. These findings indicate that AM did not promote adverse structural changes, suggesting its biocompatibility and safety when applied under normal conditions.

The AM-LPC group (Figures 1C and 2C) also presented a histological organization similar to the C–H and C-AM groups, showing no areas of demyelination or microcavitations, indicating that AM was effective in protecting neural tissue against the damaging effects of LPC, preserving the structural and functional integrity of the organotypic slices.

In contrast, in the slices belonging to the C-DEM group (Figures 1D and 2D), a significant loss of structural organization was observed, with the presence of areas of microcavitations (Figure 5), indicating damage caused by the demyelination process induced by LPC.

**Figure 5:**
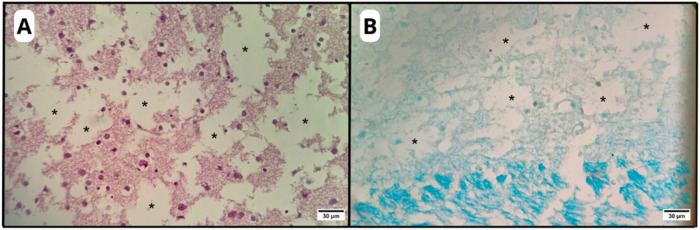
Photomicrographs of an organotypic brain slice highlighting the microcavitations observed in the C-DEM group (asterisk). HE (A) and LFB (B) stainings. Original magnification: 40×.

### Quantitative Analysis

The procedure for quantifying the total area and the demyelinated areas to obtain the preserved area was performed on three brain slices corresponding to the C-DEM group. The triplicates of the C–S, C-AM, and AM-PLC groups were analyzed only for the total area, since they did not present areas of microcavitations, remaining intact and without signs of demyelination (Table 1).

**Table 1:** Histomorphometric Data in Each of the 3 Brain Slices of the Experimental Groups

experimental group	total area (μm^2^)	demyelinated area (μm^2^)	preserved area (μm^2^)	preserved area (%)
C–H				
slice 1	7.064.319,455	0	7.064.319,455	100
slice 2	4.548.168,980	0	4.548.168,980	100
slice 3	7.465.376,187	0	7.465.376,187	100
C-DEM				
slice 1	3.074.421,719	2.059.862,552	1.014.559,167	33
slice 2	3.920.436,653	2.767.828,277	1.152.608,376	29,4
slice 3	3.024.299,976	2.080.718,383	943.581,592	31,2
C-AM				
slice 1	11.113.321,159	0	11.113.321,159	100
slice 2	6.631.408,977	0	6.631.408,977	100
slice 3	9.174.309,715	0	9.174.309,715	100
AM-LPC				
slice 1	12.113.304,927	0	12.113.304,927	100
slice 2	4.994.722,019	0	4.994.722,019	100
slice 3	10.324.056,489	0	10.324.056,489	100

After the demyelinated areas of each slice of the C-DEM group were quantified, the percentage of preserved area of the slices in relation to the demyelinated area was calculated, and the results were presented in the form of a graph (Figure 6). While the C–H, C-AM, and AM-LPC groups presented 100% of the preserved area, the C-DEM group presented an average preserved area of 31.2%.

**Figure 6:**
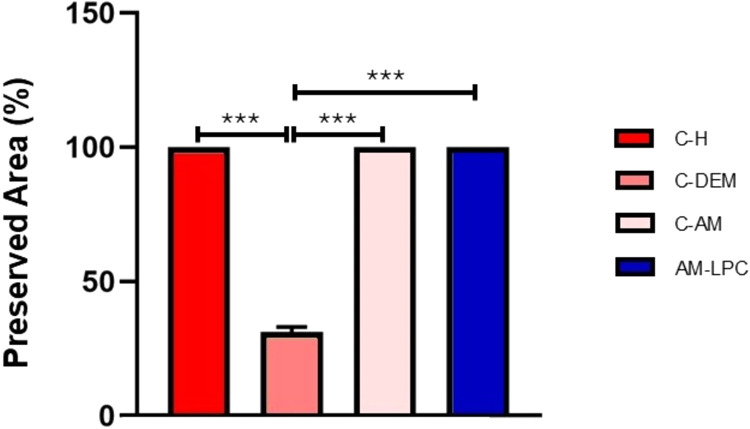
Quantitative analysis of the preservation area in experimental groups. Mean ± SD of preservation area in the experimental groups. ***p < 0.001. ANOVA and Tukey Test.

### SEM Analysis

SEM analysis provides information about the topography and surface texture of the tissue analyzed. Thus, it is possible to infer that the preservation of the uniform surface appearance is associated with the good integrity of the myelin sheath in the axons, which was observed in organotypic slices from the C–H group.

The brain parenchyma of the slices from the C–H group (Figure 7A) presented a rough surface with homogeneous characteristics and fine granulations without the presence of fissures or significant areas of discontinuity. These characteristics are indicative of healthy brain tissue, with no signs of evident structural degradation. In addition, there is no evidence of microcavitations, which suggests that the tissue layers are preserved.

**Figure 7:**
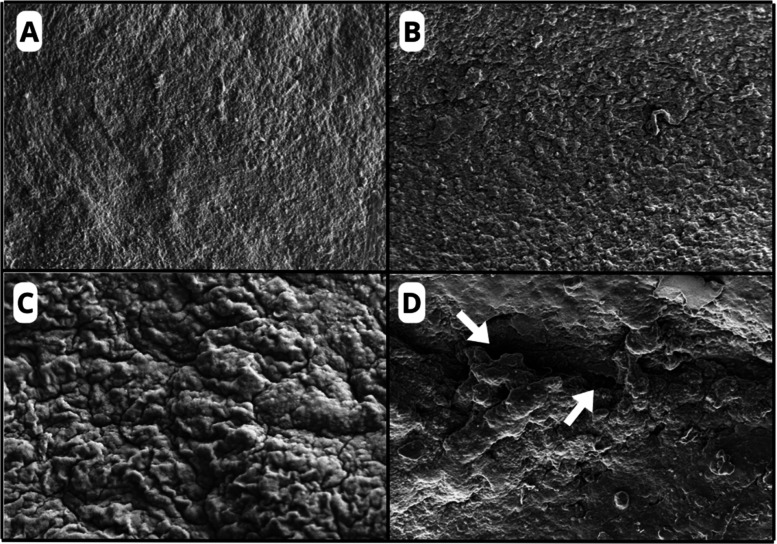
Electron micrographs of organotypic brain slices in the experimental groups: C–H (A), C-AM (B), AM-LPC (C), and C-DEM (D). Cavitations observed in C-DEM group (arrow). Magnification: 500 KX. Scale: 20 μm.

The slices from the C-AM (Figure 7B) and AM-LPC groups (Figure 7C) presented an aspect of increased surface roughness, demonstrating coarser granulations. Due to the absence of discontinuities and ruptures in the general morphology, it is suggested that the increased roughness results from an increase in the general thickening of nerve fibers in the brain parenchyma, considering the biostimulating properties of AM.

In contrast, the slices from the C-DEM group (Figure 7D) showed regions of cavitation (formation of spaces in the tissue), indicating cellular damage and structural destruction. In addition, areas with fibers that appear disordered and dispersed were also observed, which would normally be homogeneous and with well-organized fibers, as observed in the slices from the C–H group.

In the surface roughness analysis, it was observed that the C–H group presented an average standard deviation of 40.95, while the C-AM group presented an average standard deviation of 41.96. The AM-LPC group demonstrated greater roughness with a value of 51.61, followed by the C-DEM group with 50.03. These results indicate an increase in surface roughness in the AM-LPC and C-DEM groups, in relation to the C–H and C-AM groups, suggesting greater roughness in the samples subjected to demyelination and intervention with the membrane. The results of the quantitative analysis of surface roughness were presented in the form of a graph (Figure 8).

**Figure 8:**
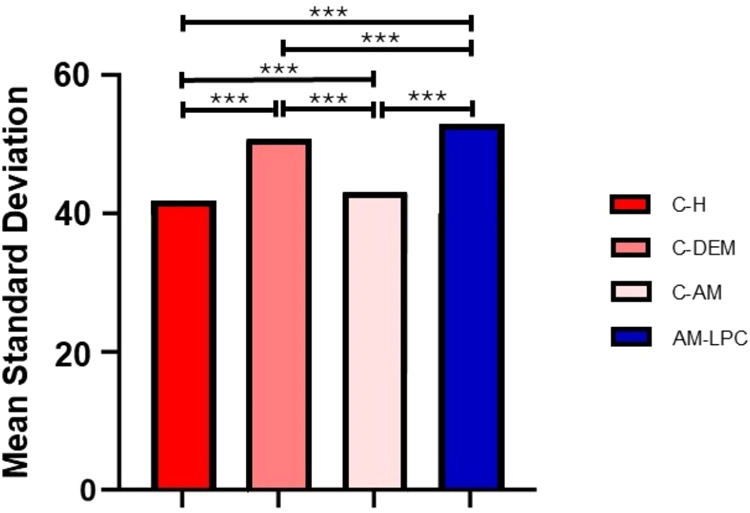
Comparative analysis of surface roughness between experimental groups: C–H, C-AM, AM-LPC, and C-DEM. Greater roughness is observed in the C-DEM and AM-LPC groups, compared to the control groups C–H and C-AM. ***p < 0.001. ANOVA and Tukey tests.

In the fissures analysis using the thresholding tool (Threshold), the percentage of dark pixels (black values) were considered as indicative of the presence of fissured areas in the images. The C–H group presented 6.25% of the dark area, while the C-AM group presented 11.37%. The AM-LPC group exhibited 20.93% and the C-DEM group presented the highest percentage, with 24.54% (Figure 9). The results of the fissure analysis, obtained through the thresholding tool, were presented in the form of a graph (Figure 10).

**Figure 9:**
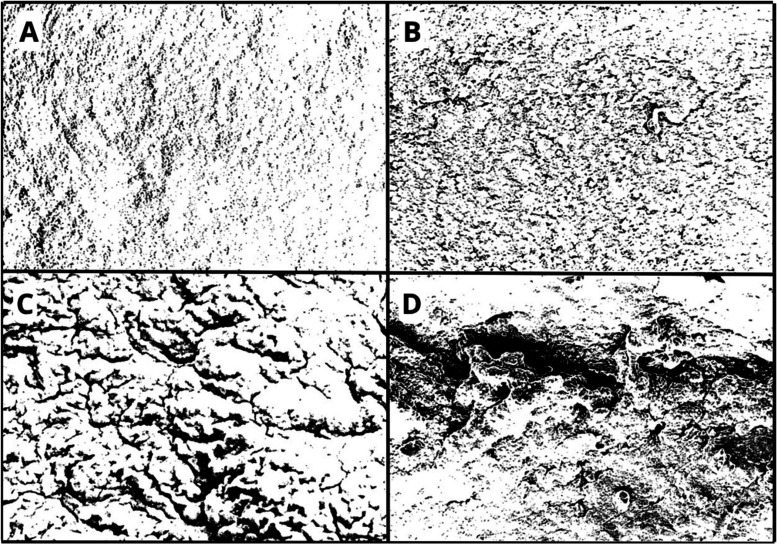
Electron micrographs of organotypic brain slices in the experimental groups after processing with the thresholding tool (Threshold): C–H (A), C-AM (B), AM-LPC (C), and C-DEM (D). The images highlight the darkened areas resulting from the processing with visible fissures in the C-DEM group (arrow). Magnification: 500 KX. Scale: 20 μm.

**Figure 10:**
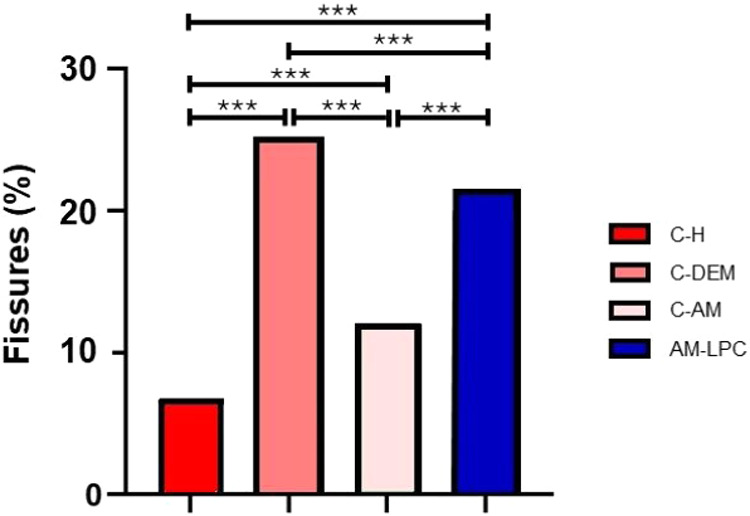
Quantitative analysis of the fissured area in the images of the different experimental groups based on thresholding by Threshold. An increase in dark pixels (indicative of fissures) can be observed in the C-DEM group compared to the C–H and C-AM groups. The AM-LPC group also presented a higher percentage of dark areas in relation to the C–H and C-AM groups. ***p < 0.001. ANOVA and Tukey tests.

In order to differentiate the greater roughness of the parenchyma from areas of fissures, a three-dimensional analysis of the surfaces was performed by using 3D graphics generated in the ImageJ software. The 3D analysis revealed that in the C–H, C-AM, and AM-LPC groups, a more uniform and elevated surface was observed, with peaks distributed in a dense and less abrupt manner, in addition to a few dark areas indicating shallower depressions. In contrast, the C-DEM group presented a more irregular surface, with a clear presence of dark regions representing deep valleys, in addition to visibly more depressed areas (Figure 11). The variation between peaks and valleys was more pronounced in this group, reflecting greater topographic unevenness and a possible compromise of the integrity of the surface structure.

**Figure 11:**
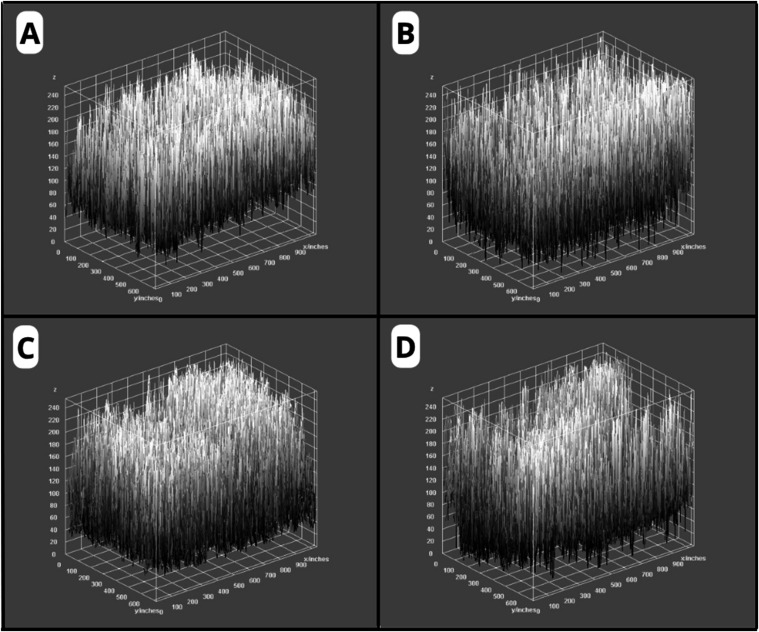
Three-dimensional representations of the tissue surface in the different experimental groups: C–H (A), C-AM (B), AM-LPC (C), and C-DEM (D). The 3D images were generated from the variation in the intensity of the grayscale pixels, reflecting the topography of the analyzed surface.

### Metabolic Activity with 2,3,5-Triphenyltetrazolium Chloride (TTC)

From the analysis with 2,3,5-triphenyltetrazolium chloride (TTC), which aims to detect cell viability, it was found that the slices of the C–H group (Figure 12A) and the C-AM group (Figure 12B) were strongly stained red, indicating preservation of cell integrity and high metabolic activity. Likewise, the slice of the AM-LPC group (Figure 12C) was also strongly stained red, indicating the preservation of metabolic activity, even when LPC was applied.

**Figure 12:**
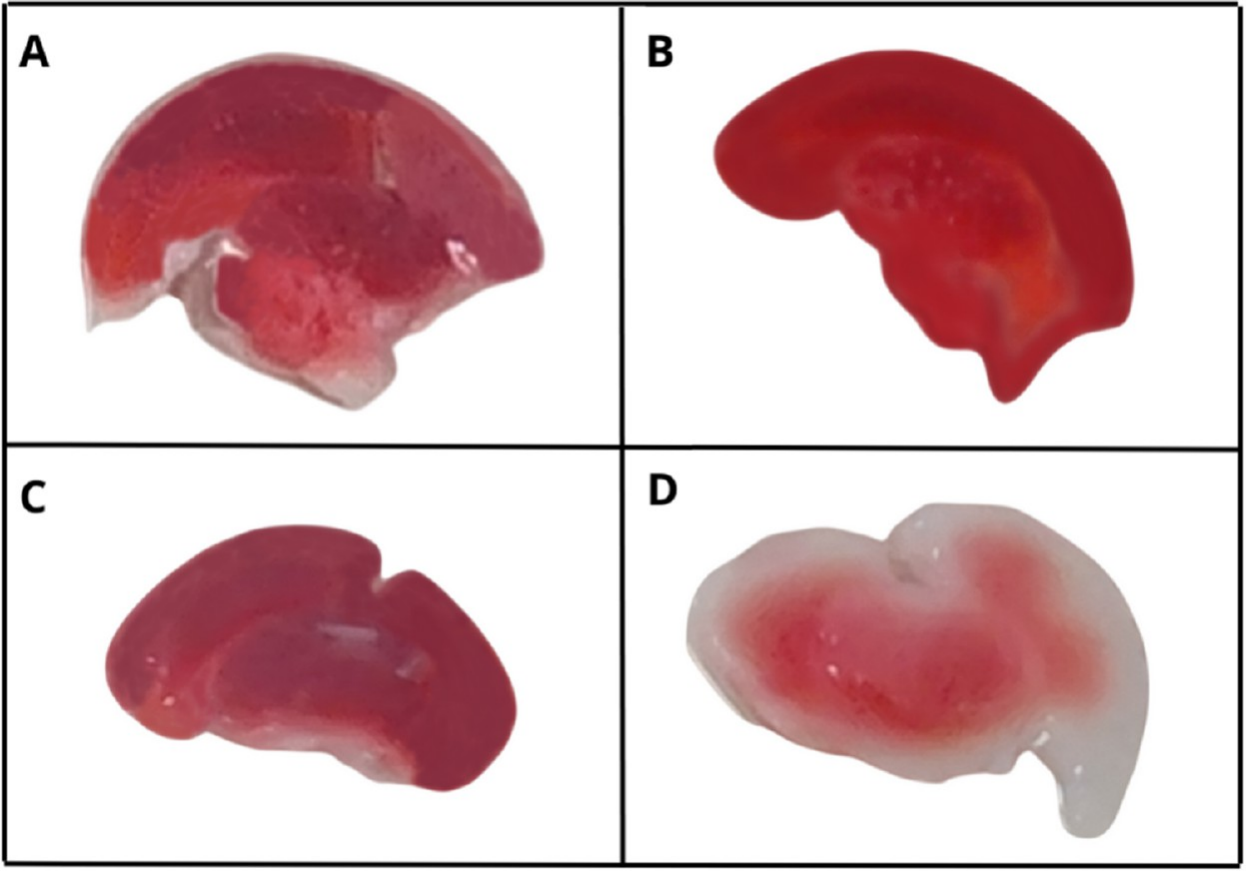
Macroscopic aspect of organotypic brain slices in the experimental groups C–H (A), C-AM (B), AM-LPC (C), and C-DEM (D), stained with TTC.

In contrast, the slice of the C-DEM group (Figure 12D) presented weaker and more irregular staining, indicating a compromised cell viability in the regions affected by LPC.

The analysis of TTC staining using the red channel in ImageJ software corroborates the qualitative analysis through images, reinforcing the visual distinction between the groups regarding tissue viability. The C–H, AM-LPC, and C-AM groups presented lower values in the mode parameter of the histogram, respectively, 139, 136, and 150, indicating intense red staining, compatible with high tissue viability. In contrast, the C-DEM group presented the highest value, 184, which indicates a significant reduction in red staining, which is compatible with lower mitochondrial activity and, therefore, lower tissue viability. The results of the TTC staining analysis were presented in the form of a graph (Figure 13).

**Figure 13:**
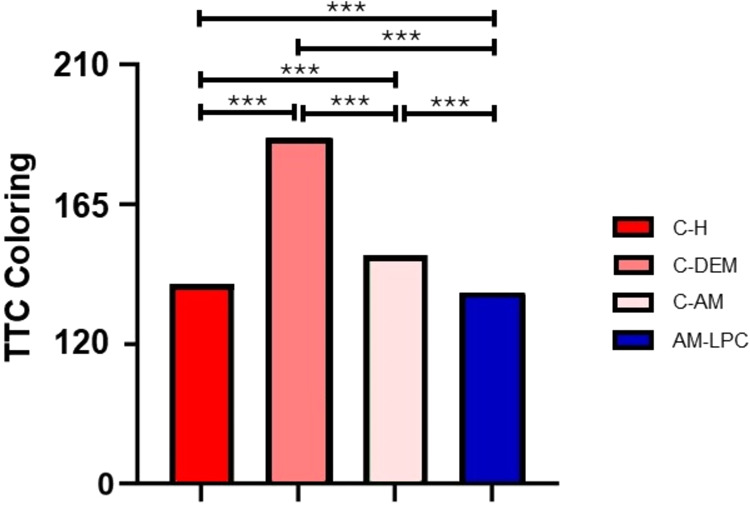
Quantitative analysis of TTC staining in different experimental groups: C–H, C-DEM, C-AM, and AM-LPC. A greater loss of viability was observed in the C-DEM group, while the C–H, C-AM, and AM-LPC groups presented more intense staining, compatible with greater cell preservation. ***p < 0.001. ANOVA and Tukey tests.

## Discussion

This study presented an innovative approach by investigating the protective potential of AM against LPC-induced demyelination in organotypic mouse brain slices, a widely used model, but still little explored using AM, since there are no studies using the amniotic membrane with this model. A particularly relevant and differentiating aspect is the fact that most of the therapeutic agents tested in this model were administered in liquid form, while AM was applied directly to the slices as a physical layer.

The histological analysis of the organotypic slices in the C–H group revealed preserved structural organization with well-defined cortical layers and a clear distinction between gray matter and white matter. The central nervous system (CNS) presents a complex and highly specialized histological organization essential for its functions. In the gray matter, molecular, granular, and pyramidal layers are observed, with the molecular layer presenting horizontalized axons and a few neurons, the granular layer rich in small granulosa cells, and the pyramidal layer containing large pyramidal neurons.^36^ This stratified organization reflects the functional integrity of normal brain tissue and corroborates the findings in the C–H group, where the cortical histoarchitecture remained preserved.

Similarly, in the C-AM group, the architecture of the brain parenchyma was also maintained with no evidence of structural damage or significant morphological changes. The white matter is characterized histologically by the predominant presence of axons surrounded by a myelin sheath. The nerve fibers may be presented longitudinally, in which it is possible to observe the axon cut along its axis or transversely. In the latter, it is possible to visualize the absence of myelin, which was dissolved during the histological preparation, and within the clear space, where there was myelin, dark structures can be observed that correspond to the transversely sectioned axons.^37^ The preservation of these histological characteristics in the C-AM group indicates that AM does not compromise tissue integrity, suggesting its biocompatibility and safety. This finding is in line with its growing recognition as a promising biocompatible scaffold for tissue engineering applications, due to its low immunogenicity, anti-inflammatory properties, and ability to support cell growth.^38^ To enhance these characteristics, researchers have explored methods to improve its mechanical properties and reduce biodegradation through cross-linking and detoxification processes.^39^ Studies demonstrate that, compared to porcine small intestinal submucosa, AM presents superior biocompatibility and greater angiogenic potential in intraperitoneal mesh repair.^40^ Furthermore, the incorporation of its bioactive macromolecules into artificial corneas reinforces its biocompatibility and antiangiogenic properties.^41^

Under conditions of demyelination, such as those experimentally induced by LPC, a significant disorganization of this histological architecture is observed. Studies have shown that injection of LPC into white matter tracts caused rapid myelin degradation, characterized by splitting of the myelin sheaths and formation of disorganized networks around axons.^42^ Furthermore, injection of LPC into the lateral olfactory tract and anterior commissure caused significant disorganization of the histological architecture, evidenced by immunohistochemistry and SEM, promoting significant demyelination within 7 days after LPC injection.^43^ Loss of myelin compromises the structural and functional integrity of the brain parenchyma, resulting in visible morphological changes, such as reduced axonal density, changes in the arrangement of glial cells, and disorganization of neuronal layers.^44^

In the present study, an analysis of HE and LFB staining revealed that, in the organotypic slices subjected to demyelination, tissue disorganization became evident, with a reduction in the preserved area of 33.2%, while the C–H, C-AM, and AM-LPC groups showed 100% tissue preservation. The parenchyma, which normally presents well-defined and recognizable histological layers, was altered, exhibiting interruptions in the continuity of these layers. A particularly relevant finding, identified through histological analysis, was the presence of microcavities in the demyelinated slices. The loss of myelin resulted in a white matter with irregularities and microcavitations, as was observed in a study with dogs with distemper, in which nervous tissue degeneration was observed, characterized by microcavitations in the brain tissue.^45^ In contrast, the slices protected by AM did not present microcavitations and maintained the histological organization of the brain parenchyma being preserved. These results suggest that AM acted as an effective physical barrier, being impermeable to the penetration of LPC, and prevented the demyelinating agent from compromising the structural integrity of the brain tissue.

AM serves as an effective barrier due to its unique ECM composition, which consists of a dense network of collagen, structural proteins, and proteoglycans, contributing to its impermeability.^17,46^ The dense and organized ECM prevents the diffusion of molecules, including demyelinating agents, which could compromise the integrity of adjacent tissues. Due to its composition of hyaluronate and other glycosaminoglycans, the ECM allows AM to act as a selective barrier, regulating the diffusion of molecules based on their charge. This electrostatic filtering mechanism allows uncharged particles to diffuse easily while trapping charged ones, contributing to AM’s hydrophobic properties, limiting the penetration of hydrophilic substances,^47,48^ enhancing its action as a selective barrier.

The interaction of LPC with lipid membranes is directly related to its ability to diffuse and interact with myelin sheaths, compromising their structural integrity. In contrast, AM has been shown to be an effective physical barrier thanks to its elasticity and mechanical resistance. Its viscoelastic properties, such as creep, stress relaxation, and elastic recovery, give it the ability to withstand significant mechanical stresses, ensuring its protective function in different biological contexts.^49–51^ Furthermore, these characteristics vary between the different regions of the AM, with the placental area being the most resistant and elastic when compared to the peripheral areas.^52^

This combination of biomechanical characteristics makes AM a fundamental element in fetal protection, by acting as a physical and physiological barrier against external agents. During gestation, the ECM of the AM undergoes physiological remodeling, a process that involves the secretion of growth factors, cytokines and matrix proteins, in order to maintain structural integrity while allowing expansion during fetal growth.^53^ Its structured layers of collagen and fetal cells ensure not only mechanical support but also immunological and endocrine functions that are fundamental for embryonic development, protecting against microbial infections and other environmental aggressions.^54–56^

In addition to its role in pregnancy, these same biomechanical and protective properties of AM have been explored for therapeutic purposes in different adult tissues. Its resistance and ability to modulate the inflammatory response have demonstrated beneficial effects in ophthalmology and dermatology, especially in the treatment of corneal ulcers, including infectious and perforating cases as well as chronic wounds. AM transplantation provides biochemical and mechanical support for corneal healing, favoring epithelialization and reducing inflammation.^57^ In addition, AM has demonstrated significant potential in the regeneration of chronic wounds, being able to modulate signaling pathways, such as TGF-β and EGF, and restore compromised healing processes, promoting re-epithelialization.^58^

The results of this study confirmed that the impermeability of AM was crucial to creating a physical barrier that prevented the direct interaction of LPC with the surface of the organotypic slices. This barrier preserved myelin in addition to maintaining the histological and functional architecture of the tissue. These findings suggest that AM not only acts as an effective protection against demyelinating agents but also contributes to the preservation of the structural integrity of the brain parenchyma under experimental conditions induced by LPC.

However, in the context of MS, AM has a therapeutic potential that goes beyond its properties as a physical barrier since it has structural and functional characteristics that are not merely passive, such as the presence of bioactive soluble factors. These are molecules, such as growth factors and ECM proteins, that are released by AM into the extracellular environment, being capable of modulating cellular and inflammatory responses that can influence the regeneration process and cell viability.^59–61^ Thus, it is suggested that these soluble factors can act directly on the neuronal microenvironment, modulating inflammation and reducing damage to the brain parenchyma, Thus, it is suggested that these soluble factors may act directly on the neuronal microenvironment, modulating inflammation and reducing damage to the brain parenchyma, potentially having an active neuroprotective role. Studies demonstrate that AM-derived mesenchymal stem cells are capable of secreting a set of bioactive molecules, known as secretomes, which act multifactorially on injured neural tissue. The medium conditioned by these cells significantly reduced infarct volume, cerebral edema, and the expression of apoptotic markers in a model of focal cerebral ischemia, indicating a direct protective effect on neurons.^62^ Furthermore, placental secretome has demonstrated protective effects in models of acute brain injury, modulating the inflammatory microenvironment, reducing secondary damage, and promoting tissue repair through the release of bioactive molecules, the secretome, with anti-inflammatory, antioxidant, and pro-regenerative actions.^63^ Additionally, human amniotic stem cells exhibit immunomodulatory, antioxidant, and proneurogenic properties, in addition to promoting vascular stability and the expression of neurotrophic factors. These mechanisms may contribute to the preservation of neural integrity and promote regeneration in demyelinating diseases such as MS.^64^ Thus, although the present study did not directly explore these pathways, the observed effects suggest that, in addition to its barrier function, AM may play an active role in the protection of neural tissue, possibly mediated by these mechanisms already described in the literature.

Despite the promising properties of AM, it was not possible to observe remyelination of the organotypic slices in this study during the established experimental period. The remyelination process involves oligodendrocyte progenitor cells (OPCs) proliferating, migrating to demyelinated areas and differentiating into mature oligodendrocytes to form new myelin sheaths.^65^ This process requires time for OPCs to progress through these stages.^66,67^ Therefore, future studies involving cultures for longer periods so that the progenitor cells can differentiate into oligodendrocytes would be necessary to verify the ability of AM to promote remyelination in the model used.

Although the histological analysis performed in this study did not aim to directly evaluate remyelination since the objective was to verify whether AM could protect the organotypic slice from LPC infiltration, a topographic analysis of the tissue surface performed by SEM revealed additional aspects of the action of AM on the nervous tissue. It was observed that the slices from the AM-LPC and C-DEM groups presented a general appearance of thickening of the nerve fibers in the brain parenchyma, unlike the C–H and C-AM groups, which exhibited a finer roughness.

This difference was confirmed quantitatively through surface roughness analysis, which demonstrated significantly higher values in the AM-LPC (51.61) and C-DEM (50.03) groups compared with the control groups C–H (40.95) and C-AM (41.96). Statistical analysis indicated that all groups differed from each other in a statistically significant manner, but the most pronounced difference was between the AM-LPC and C-DEM groups in relation to the C–H and C-AM groups. This increase in roughness may be related to changes in the parenchymal architecture caused by both demyelination and the response to AM intervention.

Furthermore, fissures analysis, based on thresholding by Threshold, indicated a higher percentage of dark areas in the AM-LPC groups (20.93%) and, especially, in the C-DEM group (24.54%), in relation to the C–H (6.25%) and C-AM (11.37%) groups. These dark areas were initially interpreted as possible fissures, that is, structural discontinuities in the tissue. However, it is important to consider that the thresholding tool used operates on 2D images, which limits its ability to differentiate true fissures from superficial porosities. While fissures imply marked recesses and slopes in the tissue, porosity may present a similar appearance in the 2D image, without necessarily representing a rupture or deep structural failure.

To overcome this limitation, a three-dimensional analysis of the surfaces was performed using 3D graphics generated in ImageJ software, allowing for more precise observation of the tissue topography. This approach revealed that although the C-DEM group presented a visibly more irregular surface, with deep regions and depressed areas (characteristics consistent with fissures), the AM-LPC group showed a more homogeneous surface, with smooth elevations and densely distributed peaks, without abrupt depressions. Thus, the signals initially interpreted as fissures in the AM-LPC group were more compatible with areas of greater porosity, associated with parenchymal thickening and tissue response to the presence of the membrane, and not with real fissures.

This finding can be interpreted as a result of a possible bioactive stimulus of AM on the brain parenchyma. Among the possible actions of this bioactive stimulus are the promotion of oligodendrocyte precursor cell (OPC) differentiation, which contributes to myelin sheath formation, and the stimulation of pre-existing oligodendrocytes to enhance their functional activity. Previous studies have demonstrated that neuronal stimulation, such as optogenetics in mouse motor cortex neurons, promotes OPC proliferation, increases oligodendrocyte differentiation, and enhances myelination in cortical and subcortical regions.^68^ In demyelinating conditions, moderate neuronal stimulation may play a significant role in inducing OPC differentiation and favoring functional myelin repair.^69^ In this context, studies demonstrate that amniotic epithelial cells have the ability to modulate the neural microenvironment through the secretion of growth factors that promote anti-inflammatory effects, favoring oligodendrocyte survival and functionality. Furthermore, the combination of AM-derived factors with sphingosine-1-phosphate (S1P) receptor modulators, such as ponesimod, has been shown to reduce oligodendrocyte apoptosis and increase their viability, suggesting a more conducive environment for remyelination.^70^

Some studies aim to evaluate the therapeutic effects of isolated AM cells on experimental models of autoimmune neuroinflammatory diseases such as experimental autoimmune encephalomyelitis (EAE). Studies have shown that human amnion mesenchymal cells (hAMC) significantly reduced the severity of EAE in mice by inhibiting the production of pro-inflammatory cytokines (IFN-γ, TNF-α, IL-1β and IL-17A) and decreasing the presence of CD4+ and CD8+ T cells in the CNS. Furthermore, the application of hAMC increased the production of neurotrophic factors (NGF, CNTF, and BDNF), favoring neuronal regeneration.^71^ Similarly, human amnion epithelial cells (hAECs) demonstrated immunomodulatory properties, suppressing the proliferation and activation of T cells, reducing the production of IL-17A and promoting the expansion of regulatory T and Th2 cells. *In vivo*, treatment with hAECs not only inhibited the development of EAE but also prevented its recurrence, reinforcing its role in regulating the immune response.^72^

The findings of the present study, obtained through histological, metabolic, and SEM analyses, indicate that the direct application of AM to nervous tissue was effective as a physical barrier against LPC-induced demyelination and in preserving brain tissue architecture. These effects suggest that, in addition to its mechanical function, AM actively modulates cellular processes in the neural microenvironment, possibly through the release of bioactive factors,^73^ which is not observed with inert materials. This application highlights the versatility of AM, demonstrating its effectiveness both as a physical barrier and as a source of bioactive factors, highlighting the importance of biomaterials in reconstructing the natural cellular microenvironment, providing mechanical support and signals for differentiation.^74^

This hypothesis is consistent with established evidence in the literature, which attributes a relevant role to the AM secretome, composed of anti-inflammatory cytokines, growth factors, extracellular vesicles, and microRNAs, in modulating the injured tissue environment.^75–77^ Studies demonstrate that these components act in a coordinated manner to attenuate inflammation, promote neuroprotection, cell regeneration, and myelin repair, as evidenced, for example, by the neuroprotective effects of the ST266 secretome in experimental models of MS.^76,77^ However, it is observed that AM presents regional heterogeneity in its morphology, marker expression, and differentiation capacity, in addition to producing extracellular vesicles in a variable manner between its different regions, which may influence its therapeutic efficacy.^78^ Furthermore, perinatal derivatives, such as multipotent stem cells and their products, have demonstrated great potential in regenerative medicine, acting primarily through paracrine mechanisms that modulate the tissue microenvironment and promote regeneration, although the exact mechanisms are still poorly understood.^79^ In this sense, the secretomes derived from these cells contain important bioactive factors, such as growth factors and cytokines, which promote essential processes for healing and vascular stabilization, reinforcing the biological basis for the use of AM in regenerative therapies.^80^ Therefore, although this study did not directly assess the molecular mechanisms involved, our experimental data align with a growing body of evidence pointing to the active role of AM as a source of signaling molecules capable of modulating the neural microenvironment and contributing to tissue preservation. These findings strengthen the hypothesis that the observed protection is due not only to the physical insulation provided by AM but also to the presence of intrinsic bioactive properties that differentiate it from inert materials.

In addition to the action of bioactive factors, another relevant factor is the mechanical influence of the ECM of AM, which provides physical and biochemical support, facilitating axon regeneration and functional recovery in peripheral nerve injuries.^81^ Its structure mimics the native microenvironment, offering topographic and chemical cues that guide the formation and reconstruction of neural tissue.^82^

Furthermore, corroborating the histomorphometric and SEM findings, the analysis of TTC staining showed relevant differences in the cell viability between the experimental groups. Quantification, performed in the red channel using ImageJ software, demonstrated that the C–H and C-AM groups presented lower values in the mode parameter of the histogram (139 and 150, respectively), indicating more intense red staining. This staining is associated with the presence of preserved mitochondrial activity, suggesting high tissue viability and cell integrity in these groups. The C-DEM group presented a significantly higher mode value (184), reflecting a lower intensity of red staining. This finding is consistent with reduced mitochondrial activity and impaired cell viability resulting from the demyelinating action of LPC. In contrast, slices from the AM-LPC group presented intense staining, with lower values in the mode parameter of the histogram (136), suggesting that AM was effective in protecting the tissue against the demyelinating action of LPC, maintaining cell viability.

The ability of AM cells to increase cell viability was quantitatively evaluated in a study in which the ST266 secretome, a set of molecules secreted by multipotent progenitor cells derived from MA, showed neuroprotective and remyelination-promoting effects in the EAE model. Intranasal administration of ST266 improved retinal ganglion cell survival, as assessed by the WST-8 Cell Quantification Assay, and reduced optic nerve demyelination. Furthermore, the therapeutic effects were more evident when high molecular weight proteins of the secretome were preserved, suggesting that these components play an essential role in neuroprotection and remyelination.^83^ In contrast, as observed in this study, the slices of the C-DEM group exhibited weaker and more irregular staining, evidencing compromised cell viability in the affected areas, possibly reflecting the loss of the structural and functional integrity of the tissue.

The application of AM in the experimental model of organotypic brain slice culture used in this study highlights the versatility of AM, demonstrating its efficacy both as a physical barrier against adverse agents and as a source of bioactive factors that act in the modulation of the neural microenvironment. Thus, AM proves to be a promising alternative in the therapy of demyelinating diseases, such as MS. However, to validate this applicability, additional studies are needed using experimental models that simulate the autoimmunological mechanisms of MS, to investigate how AM influences the specific immunological processes of this condition. These studies are essential to determine whether the protective and biostimulatory effects of AM, already observed in this work, may be relevant in scenarios in which an exacerbated immune response plays a central role in demyelination. Although the present study does not directly address the immunological mechanisms involved in MS, it demonstrates that due to the constitution of its ECM and the bioactive factors it releases, AM has promising therapeutic potential in demyelinating diseases, such as MS.

## Conclusions

It is concluded that from the results obtained in this study, AM was effective in functioning as a protective agent against LPC-induced demyelination in organotypic brain slices of mice. In addition to acting as an effective physical barrier, AM showed bioactive properties that favor the preservation and integrity of nervous tissue, revealing itself as a promising alternative in demyelinating diseases.
